# Learning to find spatially reversed sounds

**DOI:** 10.1038/s41598-020-61332-4

**Published:** 2020-03-12

**Authors:** Fernando Bermejo, Ezequiel A. Di Paolo, L. Guillermo Gilberto, Valentín Lunati, M. Virginia Barrios

**Affiliations:** 1 Centro de Investigación y Transferencia en Acústica, Universidad Tecnológica Nacional - Facultad Regional Córdoba, CONICET, CP 5016 Córdoba, Argentina; 2 Facultad de Psicología, Universidad Nacional de Córdoba, CP 5016 Córdoba, Argentina; 3 Ikerbasque, Basque Foundation for Science, Bilbao, Spain; 4 IAS-Research Center for Life, Mind, and Society, University of the Basque Country, San Sebastián, Spain; 5 Centre for Computational Neuroscience and Robotics, University of Sussex, Brighton, UK; 6 Consejo Nacional de Investigaciones Científicas y Tecnológicas (CONICET), Ciudad Autónoma de Buenos Aires, Argentina

**Keywords:** Auditory system, Human behaviour

## Abstract

Adaptation to systematic visual distortions is well-documented but there is little evidence of similar adaptation to radical changes in audition. We use a pseudophone to transpose the sound streams arriving at the left and right ears, evaluating the perceptual effects it provokes and the possibility of learning to locate sounds in the reversed condition. Blindfolded participants remain seated at the center of a semicircular arrangement of 7 speakers and are asked to orient their head towards a sound source. We postulate that a key factor underlying adaptation is the self-generated activity that allows participants to learn new sensorimotor schemes. We investigate passive listening conditions (very short duration stimulus not permitting active exploration) and dynamic conditions (continuous stimulus allowing participants time to freely move their heads or remain still). We analyze head movement kinematics, localization errors, and qualitative reports. Results show movement-induced perceptual disruptions in the dynamic condition with static sound sources displaying apparent movement. This effect is reduced after a short training period and participants learn to find sounds in a left-right reversed field for all but the extreme lateral positions where motor patterns are more restricted. Strategies become less exploratory and more direct with training. Results support the hypothesis that self-generated movements underlie adaptation to radical sensorimotor distortions.

## Introduction

When confronted with devices that affect visual perception, human adults can recalibrate sensorimotor patterns and compensate, at least partially, for substantial systematic distortions such as a left-right reversion of the visual field. Even when adaptation is not complete, these cases offer clues for understanding general principles common to all sensory modalities^1–4^. Adaptation to radical visual changes is well-documented. However, similar evidence for other modalities is scarce. In this paper we show evidence of adaptation to a left-right reversion of the auditory field in a sound localization task.

Several studies show adaptation to radical visual distortions using prisms or mirrors that exchange spatial directions left and right or up and down, or induce systematic angular displacements and curvatures of the visual image^1,5–7^. Wearing inversion/reversion goggles brings about unexpected visual changes as participants move, making them feel baffled at first. One reason for this is that extra-retinal proprioceptive signals cease to compensate for the retinal flows provoked by the static visual background as the body moves. This lack of habitual compensation gives rise to perceived visual flows in unexpected directions. As participants gradually adapt, the effects of movement-induced instability diminish.

In spatial hearing there are fewer attempts to study adaptation to similar radical changes^8–11^. Instead of goggles, a pseudophone is used in these cases. The pseudophone is a device that transposes the sound streams reaching the left and right ears. Studies looking at relatively short periods of use do not show adaptation to the pseudophone. In many cases, this may be due to a dominance effect of vision over hearing. If during training, participants are exposed to visible sound sources, they can disregard auditory spatial information and resolve any perceptual conflict through the ventriloquism effect^12^. As in vision, perceptual instability is also manifest as participants move themselves in a reversed acoustic field. In some studies participants can move the head voluntarily and report that as they move (static) sound sources also seem to move^8,11^.

Adaptation can occur for less radical angular displacements. Richard Held^13^ studied the effects of 22 degree clockwise or counterclockwise displacements on the ability to locate sounds. He evaluated 6 participants before, during, and after 1 hour of exposure. Participants were blindfolded. They could adapt if allowed to approach and move away from the sound source on their own. Held suggested that adaptation takes place when participants are able to establish an association between a given interaural difference and the particular movement their body needs to make to move toward the source.

Finding sounds on the horizontal plane depends on the differences in time and intensity with which sounds arrive at the ears^14,15^. The pseudophone distorts these interaural cues with effects that depend on motor activity. Perception of a stable auditory world implies mastering a body-centered coordinate system. This system integrates information about the body’s position with respect to sound sources^16,17^ and the patterns of sound variations generated as the body moves^18^. Studies of spatial hearing emphasize the role played by voluntary movement during adaptation, such as in the suppression of one auditory channel^19^, pinna modification^20^, hearing impairment^21^, and the use of bilateral cochlear implants^22,23^. In line with theories that highlight the active nature of perception^24,25^, sound localization learning has been modelled on the basis of the sensory consequences of voluntary motor actions^26^. The accumulated evidence supports the view that auditory space, like visual space, is structured by active sensorimotor engagements performed by the perceiver^25^.

If motor activity is important for rebuilding disrupted sensorimotor relations and regaining some degree of stable perception, then we should expect adaptive success to depend on the availability of motor patterns during the adaptation period. In this paper we test this idea in a sound localization test using a left-right reversion pseudophone. We ask whether participants can learn to locate a source of sounds while using a pseudophone and the extent to which less restricted motor patterns lead to better adaptation. Following the protocol used by Held^13^, blindfolded participants wearing a pseudophone and a head motion tracker are asked to orient towards a sound source while sitting at the center of a semicircular arrangement of 7 speakers located at face height. Participants can move their heads freely. They are asked to give their response by orienting the face toward the position where they perceive the sound to be coming from. We test for learning effects in relation to source position and motor strategies, taking into account errors in localization, head movements, and qualitative information from self-reports.

## Methodology

### Participants

Twenty adults participated in two experiments (ten each). They reported no hearing problems and none had previous knowledge of the experimental set-up. The study was carried out in accordance with the Helsinki Declaration guidelines. The protocol received institutional approval from Universidad Nacional de Córdoba. All participants provided written informed consent prior to beginning the experiment.

### Experimental set-up

**Figure 1:**
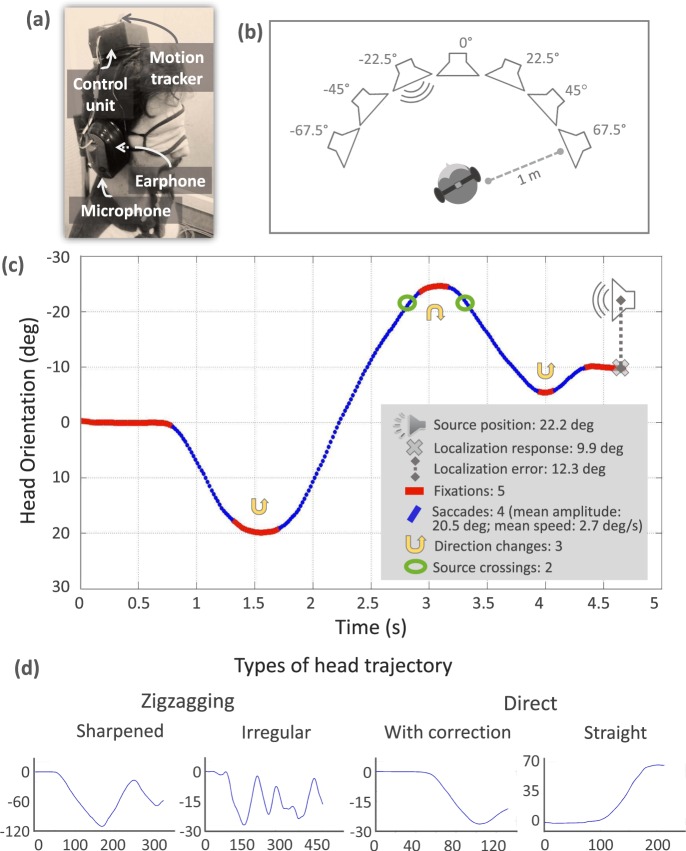
Experimental setup and data analysis. (**a**) Photo of one of the authors (F.B.) wearing the pseudophone. (**b**) Diagram of the experimental array. (**c**) Example of a head movement trajectory of a trial showing the parameters evaluated for data analysis. The vertical and horizontal axis represent the head orientation and time, respectively. (**d**) Real examples of trajectories of different types.

The pseudophone is fitted with an earmuff to attenuate sounds that reach each ear directly. Each cup has a small speaker on the inside and a microphone on the outside. A control unit is attached to the headband allowing the experimenter to reverse (reversed mode) or not (normal mode) the acoustic signals and also to change the amplifier gain. In the reversed mode the speakers reproduce the signal picked up by the contralateral microphone. The operation of the device was tested with different electroacoustic measurements. Sound attenuation and insertion losses (according to EN 352-1) were measured with average values greater than 32 dB and 20 dB respectively. The symmetry of the electronic circuits was verified and the ITD and ILD were measured. Other details of the device appear in a previous publication^27^. A motion tracker (Polhemus Patriot) mounted on the pseudophone registers head movements (Fig. 1a).

We performed the experiments in a semi-anechoic chamber of dimensions 4.20 × 3.80 × 2.60 m, with a reverberation time of 170 ms (measured at 1 kHz octave band) and 17 dBA SPL background noise.

Seven speakers are arranged in a semicircle with the participant at its center (Fig. 1b). Their positions: Left Lateral −67.5 deg, Left Intermediate Lateral −45 deg, Ahead to the Left −22.5 deg, Central 0 deg, Ahead to the Right 22.5 deg, Right Intermediate Lateral 45 deg, and Right Lateral 67.5 deg. For participants these positions are not exact but approximate because their movements alter the relative position of the source with respect to their heads. These angular modifications are taken into account to constantly recalculate the source position according to the actual position of the head from the beginning to the end of trials.

The participant sits in a chair located in the center of the array at 1 m distance from the speakers (Fig. 1b). Speakers are placed at 110 cm from the floor, at the level of the participant’s face. The sound stimulus is pink noise bandpass-filtered between 0.3 kHz and 9 kHz, its level was 70 dB SPL measured at 1 m from the source. Stimulus duration varies according to the experimental condition, as described below. Stimuli generation and reproduction is controlled through an 8-channel system, based on a field-programmable gate array. The test management software developed in an application based on MATLAB GUI allows to synchronously coordinate the sound reproduction and data acquisition from the motion tracker via serial port.

### Experiment 1: Disruptive effects

We measured the disruptive effect of the pseudophone on first use in passive and dynamic listening conditions. The participant, blindfolded and wearing the pseudophone, remains seated. At the beginning of each trial they face the center of the arrangement. When a sound source is activated, the participant’s task is to explore the environment with rotational head movements and point toward the sound source with the nose. To give the localization response they remain still and indicate it verbally to the experimenter.

We evaluated the performance of 10 participants (5 female, Mean age: 23.5, SD: 1.9) in 4 different listening conditions:

- Normal Passive: pseudophone in normal mode and the stimulus a brief (120 ms) pulse of pink noise. The brevity of the stimulus prevents participants from using self-generated dynamic cues^28^.
- Normal Dynamic: pseudophone in normal mode and the sound source remains active with uninterrupted pink noise until participants give their answer. They are able to actively rotate the head or remain passively still at any orientation.
- Reversed Passive: As Normal Passive but with pseudophone in the reversed mode.
- Reversed Dynamic: As Normal Dynamic but with pseudophone in the reversed mode.

Two researchers performed the experiment, one controlled the software, while the other was responsible for reading instructions and interacting with the participant. In total, each participant completed 196 trials (4 listening conditions × 7 positions × 7 repetitions). They were allowed to rest briefly after completing each experimental condition. A session took approximately 50 minutes. The order of presentation of listening conditions was: (1) Normal Passive; (2) Normal Dynamic; (3) Reversed Passive; (4) Reversed Dynamic. The order of presentation of source positions was different for each participant according to randomly defined protocols. After each session, we conducted a semi-structured interview to collect information of participants’ experience.

### Experiment 2: Perceptual learning

We test for learning when the auditory field is reversed. A different set of 10 participants (4 female, Mean age: 23.8, SD: 2.4) performed the same test as in the previous experiment in the dynamic listening conditions, that is, the sound signal was always uninterrupted pink noise until participants gave their response. Participants completed 2 series of trials in normal and reversed listening conditions to obtain a baseline status of their skills, then a training phase with feedback on performance, and finally 2 more series in randomly presented normal and reversed listening conditions. In detail:

- Normal 1: pseudophone in normal mode without performance feedback.
- Reversed 1: pseudophone in reversed mode without performance feedback.
- Training in reversed condition: pseudophone in reversed mode with performance feedback. After each trial the experimenter verbally indicates “correct answer” when the participant’s head points to the active source (±5 deg); “in the same direction” when the head points near the active source (±22.5 deg); and “incorrect” when the head points away from the active source (>22.5 deg). Participants undergo at least 28 trials (7 positions × 4 repetitions). To move on to the next listening conditions, participants have to respond correctly in at least 5 of the last 7 trials.
- Normal 2: pseudophone in normal mode with the same performance feedback as in training.
- Reversed 2: pseudophone in reversed mode with the same performance feedback as in training.

The last two listening conditions are administered randomly during the final phase without informing participants about the pseudophone setting. This is done to avoid the use of “shortcut strategies” where the participant simply starts by choosing the opposite direction to the source they listen to if they know the listening condition is reversed. This had been observed in pilot studies. In these last trials we continue giving feedback because the mixed presentation of conditions increases the complexity of the task.

Two researchers performed the experiment. Not counting the training phase, each participant completed 112 trials (4 listening conditions × 7 positions × 4 repetitions). Participants could rest briefly before training trials. The test took approximately 90 minutes. The order of trials was: (1) Normal 1; (2) Reversed 1; (3) Training; (4) random selection of Normal 2 and Reversed 2. The order of presentation of source positions was different for each participant according to randomly defined protocols. After each session, we conducted a semi-structured interview to collect information of participants’ experience.

### Data analysis

We recorded the localization responses, i.e., the final head orientation in degrees on the horizontal plane, taking as response bias the localization error, i.e., the absolute difference expressed in degrees between the localization response and the source position. We analyzed head movements to calculate the amount of direction crossings on the active source, exploration trajectories, and movement indexes. For this, we adopted a technique used for studying ocular saccades and fixations^29^. This analysis allows the identification of periods of relative quiet, analogous to visual fixations, and discretizes the trajectory of movements in a series of minor movements, analogous to visual saccades. Based on this analysis we refer to head movements as “head saccades”. We obtained parameters for each head saccade such as amplitude, speed, and orientation, and calculated changes in direction (Fig. 1c).

From these results we identified different types of localization strategies. Trajectories can be **Zigzagging** or **Direct**. We evaluated the number of direction changes in each trajectory. If there is more than one direction change the trajectory is zigzagging, if there is none, it is direct. When there is only one direction change, we look at the angle traced by the two head saccades, if this is greater than 22.5 deg the trajectory is considered as zigzagging; if it is less, as direct. Depending on the change in the amplitude of the head saccades a zigzagging trajectory can be: **Sharpened** when the amplitude is progressively smaller; or **Irregular**, when the amplitude varies without apparent pattern. This classification is determined by evaluating the tendency of successive head saccades amplitude using the least square method. We calculate the linear function that best fits with successive saccade’s data, and evaluate its slope for classifying zigzagging trajectories as: sharpened, when the slope is <=−3 (observable amplitude decreasing trend), and irregular, when the slope is >−3 (no observable decreasing trend). Direct trajectories are classified into **Straight**, when it is a continuous head movement or **With correction**, when a head saccade is added at the end (Fig. 1d).

To analyze the relation between source position and localization response, we first use the coefficients of the linear regression using least squares estimation: Pearson’s correlation, general gain and SD of the residual error after the regression. Pearson’s correlation (r) provides information on the strength and direction of the relation between sound source and localization response. The gain (G) is the slope of the regression line and indicates underestimation and overestimation patterns in the responses. The sign provides information on the direction of the linear relationship. The SD of the residuals of the fitted function (δ) indicates the distribution of responses in relation to G and gives an estimate of imprecision in the localization responses. Then, we analyze the localization errors, the absolute difference between source position and localization response, to compare the effects of listening conditions and source positions.

Data on localization errors, exploration strategies and head saccade parameters are first tested for normality using the Shapiro-Wilk test. To compare the effect of source position and listening condition, in the case of a normal distribution, we perform related samples t-test and repeated measures analysis of variance (ANOVA) followed by Newman–Keuls post-hoc comparisons. In the case of non-normal data sets we apply Wilcoxon signed-rank tests and Friedman test followed by Wilcoxon signed-rank tests pairwise comparisons. In all cases a p-value of 0.05 is considered as statistically significant.

## Results

### Experiment 1: Disruptive effects

We measure the disruptive effect of the pseudophone on first use to determine differences between passive and dynamic listening conditions. We evaluate 10 participants in 4 listening conditions: Normal Passive, Normal Dynamic, Reversed Passive, and Reversed Dynamic. Results show movement-induced disruption only in the Reversed Dynamic condition. In this condition, most participants show unpredictable changes in the accuracy of the responses. They often switch between exploration strategies and report experiences of disorientation.

#### General performance

**Figure 2:**
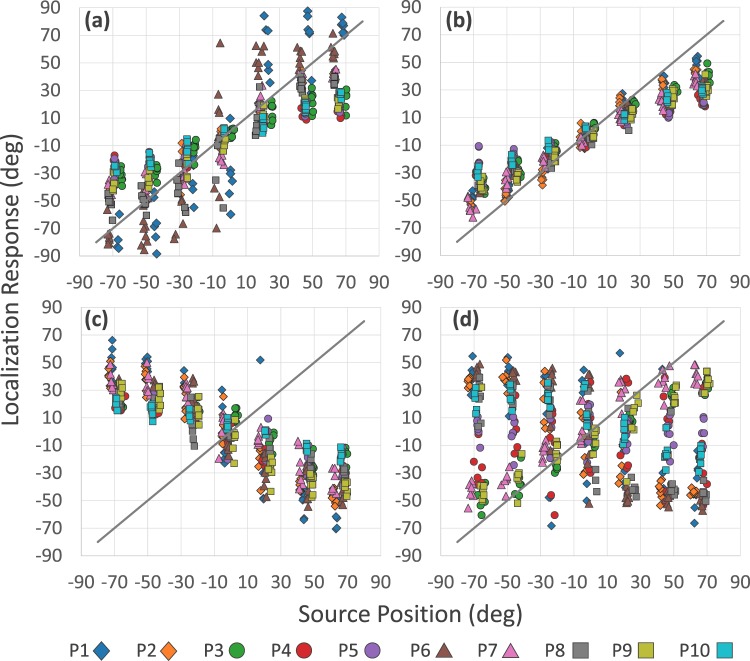
Distribution of localization responses in Experiment 1 according to listening condition: (**a**) Normal Passive, (**b**) Normal Dynamic, (**c**) Reversed Passive, and (**d**) Reversed Dynamic. Each participant’s data (P1 - P10) is shown with different symbols.

Figure 2 and Table 1 give an overview of the performance of the participants. In normal listening conditions, due to interaural distance and head-related transfer functions being modified by the device, sound localization is not perfect with low gains. This results in a larger response variability than is typically obtained in absolute free-field soundlocalization tasks. However, response patterns are similar to those found in typical studies that use head orientation as the response method^30^. In general, performance is better in the frontal region than at the periphery, where participants tend to underestimate the position of the source. In the Dynamic Normal condition performance is better compared to Passive Normal. Levels of r are higher, G is closer to 1 and values of δ are lower. Probably, this is because in the Normal Dynamic condition participants have more information to refine their response, as shown previously^31^. In the Reversed Passive condition performance also fits with a linear regression. The values of r and δ are similar to those found in Normal Passive. However, all G values are negative, that is, in this listening condition the slope of the regression is negative. Responses are mirrored in the opposite hemifield to the sound source. In the Reversed Dynamic condition, in general, negative G values are maintained but r decreases indicating a worse association between responses and the position of the source. Values of δ increase suggesting that responses are more imprecise. In addition, in the latter condition 3 participants (P3, P7 and P9) obtained similar results to those they had in normal listening conditions: high values of r, relatively low values of δ and positive G values, indicating that their localization responses were in the hemifield where the source was, that is, their responses were not reversed. To clarify the effect of listening conditions on most participants, we exclude the data in these 3 cases in the following comparisons and consider them separately at the end of the section.

**Table 1:** Linear regression coefficients for each participant in Experiment 1.

	Normal Passive			Normal Dynamic			Reversed Passive			Reversed Dynamic		
	r	G	δ	r	G	δ	r	G	δ	r	G	δ
P1	0.89	1.21	28.06	0.99	0.76	5.61	0.93	−0.90	16.91	0.68	−0.60	29.40
P2	0.98	0.73	7.39	0.98	0.79	6.75	0.96	−0.73	9.95	0.90	−0.68	15.50
P3	0.96	0.47	6.47	0.99	0.61	4.06	0.91	−0.43	8.91	0.98	0.63	5.92
P4	0.94	0.32	5.38	0.99	0.42	3.21	0.96	−0.41	5.41	0.42	−0.11	11.13
P5	0.97	0.37	4.42	0.97	0.32	3.61	0.90	−0.39	8.48	0.09	−0.02	11.37
P6	0.91	1.20	24.81	0.99	0.56	3.41	0.93	−0.66	12.20	0.89	−0.84	20.13
P7	0.95	0.74	10.57	0.99	0.66	4.62	0.94	−0.60	9.91	0.96	0.68	9.64
P8	0.93	0.77	13.56	0.99	0.51	3.18	0.95	−0.45	6.82	0.79	−0.57	21.15
P9	0.99	0.57	4.51	0.99	0.57	4.51	0.96	−0.60	8.36	0.85	0.53	15.09
P10	0.99	0.45	2.81	0.99	0.45	2.81	0.96	−0.28	3.63	0.88	−0.38	9.48

Rows show for each participant (P1–P10) the values of coefficients of the linear fit according to the listening conditions (r: Pearson’s correlation; G: gain; δ: SD of residuals).

We perform a repeated-measures ANOVA on mean localization error using source position and listening condition as factors. There is a main effect of source position (F_(6, 36)_ = 141.29, p < 0.0001, η^2^: 0.959). The post hoc analysis shows that localization errors are significantly higher at the Lateral positions, right and left, than at the rest of the positions (p < 0.0001 for all comparisons). These are followed by errors in Intermediate Lateral positions, right and left, which are significantly higher than in the remaining positions (p < 0.0001 for all comparisons). Errors in Ahead to the Left and Ahead to the Right positions are higher than for the central position (p < 0.0001 for both comparisons). The magnitude of localization errors increases as the source is located further away from the center. There is also a main effect of listening condition (F_(3, 18)_ = 39,019; p < 0.001; η^2^ = 0,866). Pairwise post hoc testing shows that errors in Reversed Passive are significantly higher than in Normal Passive (p < 0.0001) and in Normal Dynamic (p < 0.0001), while errors in Reversed Dynamic are significantly higher than in Normal Passive (p < 0.0001) and in Normal Dynamic (p < 0.0001). This means that participants perform better in normal listening conditions than in reversed ones. To test whether this difference is due only to an effect of the reversion, or whether there is an effect of movement in the reversed condition, we invert the sign of all localization responses in the reversed conditions. That is, if a participant indicated that the source was at “–25 deg, we impute “25 deg”. The same analysis now shows an effect of the listening condition factor (F_(3, 18)_ = 8.317, p < 0.001, η^2^ = 0.580). But this time the post hoc comparison shows that only performance in Reversed Dynamic is significantly worse than in Normal Passive (p < 0.001), Normal Dynamic (p < 0.002), and Reversed Passive (p < 0.002). This result confirms that responses in Reversed Passive are simply mirrored in the opposite hemifield, preserving a similar pattern to that of the normal listening conditions. While in Reversed Dynamic, head movements make performance more variable as pointed out in previous studies with pseudophones^10,11^.

#### Motor trajectories

**Figure 3:**
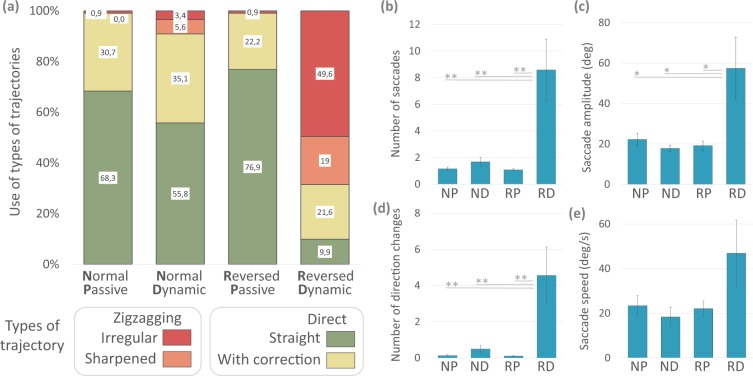
Trajectories and indexes of head movements in Experiment 1. (**a**) Percentage of use of each type according to listening condition. On the right the plots show the mean values (±SEM) of different parameters of head saccades according to listening condition (NP: Normal Passive; ND: Normal Dynamic; RP: Reversed Passive; RD: Reversed Dynamic): (**b**) shows the number of head saccades, (**c**) their amplitude, (**d**) the number of direction changes, and (**e**) their speed (*p < 0.05, **p < 0.001).

We perform Friedman tests to assess the frequency of use of each strategy according to listening condition, and Wilcoxon tests for pairwise comparisons. In the case of Direct-Straight trajectories there are significant differences (X2r = 2.803, p < 0.005). Trajectories of this type are less used in Reversed Dynamic than in Normal Passive (Z = 2.803, p < 0.005), in Normal Dynamic (Z = 2.665, p < 0.007) and in Reversed Passive (Z = 2.665, p < 0.007). We also find significant differences for Zigzagging-Sharpened trajectories (X2r = 16.529, p < 0.0008). They are more used in Reversed Dynamic listening condition compared to Normal Passive (Z = 2.366, p < 0.01) and Reversed Passive (Z = 2.366, p < 0.01). Finally, Zigzagging-Irregular trajectories are also significantly different (X2r = 18.241 p < 0.0003), being more used in Reversed Dynamic condition than in Normal Passive (Z = 2.366, p < 0.01), Normal Dynamic (Z = 2.201, p < 0.02), and Reversed Passive (Z = 2.366, p < 0.01). In sum, in listening conditions Normal Passive, Normal Dynamic, and Reversed Passive participants mostly use direct trajectories. In Reversed Dynamic condition they mainly use zigzagging trajectories (Fig. 3a).

We analyze different parameters for movements that make up each trajectory (Fig. 3b–e) and compare the mean number of head saccades per trial with a repeated-measures ANOVA taking the listening condition as a factor. We find significant effects (F_(3, 18)_ = 9.952, p < 0.001, η^2^: 0.623). The post hoc analysis shows that participants make significantly more head saccades in Reversed Dynamic than in Normal Passive, Normal Dynamic, and Reversed Passive (p < 0.001 for all comparisons). We find differences in the mean amplitude of head saccades in each trial (F_(3, 18)_ = 6.464, p < 0.01, η^2^: 0.518). Post hoc test indicates that amplitude is significantly higher in Reversed Dynamic than in Normal Passive, Normal Dynamic, and Reversed Passive (p < 0.01 for all comparisons). We also find effects in the mean number of direction changes per trial (F_(3, 18)_ 7.655 p < 0.001 η^2^ 0.560). Post hoc comparison shows that participants make significantly more direction changes in Reversed Dynamic than in Normal Passive, Normal Dynamic, and Reversed Passive (p < 0.0001 for all comparisons). The comparison of the effect of listening conditions on the mean speed of head saccades does not reach statistical significance. Together, these results indicate that saccade-like head movements in the Reversed Dynamic condition are more frequent, larger, and with more direction changes than in the rest of listening conditions.

#### Participants’ reports

Participants’ comments on their experience agree with the difficulty with the Reversed Dynamic condition evidenced in the data analysis. They report that only in this condition did they feel confused and were unsure if they had performed the task well. For example: “*The [Reversed Dynamic] condition was the hardest. I went back and forth trying to find where [the source] was*” (Participant 1); “ *[Condition Reversed Dynamic] was more difficult. I had trouble identifying where the sound was coming from. Sometimes the sound cleared up and sometimes it got lost. I don’t know how to explain it, the sound moved*.” (Participant 2).

Several participants mentioned that when they moved, so did the sound. Some claimed to perceive the source in the opposite position on the backplane, i.e., behind the participant. A similar effect has been reported before^11^, potentially explainable because the sensorimotor patterns performed in the reversed listening condition are similar to those used to locate sounds in the backplane in the normal condition.

### Experiment 2: Perceptual learning

In this experiment we test for learning when the auditory field is reversed. A different set of 10 participants performed the sound localization task in dynamic listening conditions, that is, the source remaining active until participants give their response. First, participants performed trials in normal and reversed listening conditions, Normal 1 and Reversed 1, respectively. Then, they performed a series of training trials. Finally, they performed trials in which the pseudophone, without their knowledge, was randomly set in the normal or reversed condition. We group trials during this phase under the labels Normal 2 and Reversed 2 respectively. Results shows evidence of variable adaptation to the reversed listening condition after the training. Movement-induced disruption effects observed in Reversed 1 at the beginning are reduced in Reversed 2 after training. Most participants learn to locate sounds in a left-right reversed field except at the extreme lateral positions where motor patterns are more restricted. The analysis shows the importance of motor activity for achieving adaptation.

#### General performance

**Figure 4:**
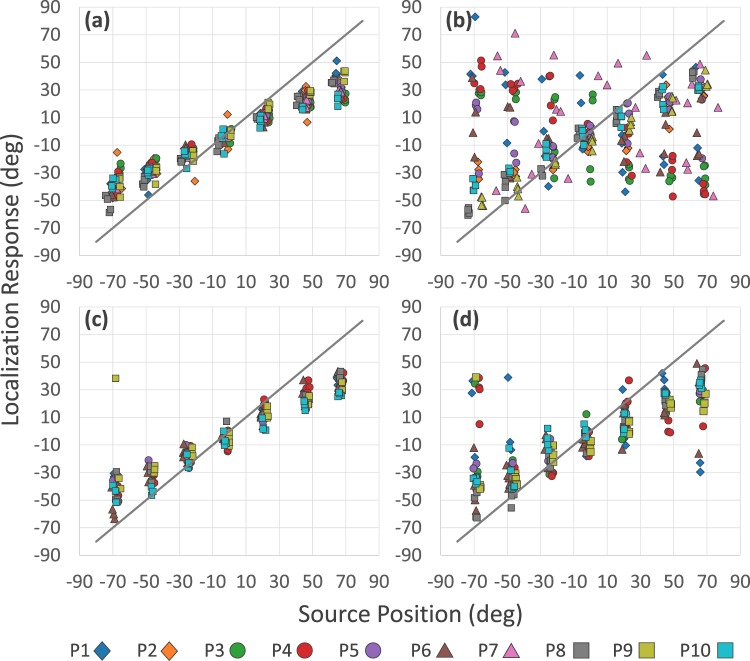
Distribution of localization responses in Experiment 2 according to listening condition: before training (**a**) Normal 1, (**b**) Reversed 1, and after training (**c**) Normal 2, and (**d**) Reversed 2. Participant’s data (P1–P10) are indicated with different symbols.

Most participants, to different degrees of accuracy, learn to locate sound sources in the reversed listening condition (Fig. 4 and Table 2). As in the previous experiment, 3 participants (P8, P9 and P10) were able to correctly locate the sound from the start in the reversed condition, their values of G are positive and also, the values of r and δ are similar to those obtained in Normal 1. Accordingly, these participants did not perform the training phase. Two participants (P2 and P7) could not learn to locate sounds in the reversed condition and did not complete the second series of trials. Their data were excluded from the following analyses and are described at the end of the section.

**Table 2:** Linear regression coefficients for each participant in Experiment 2.

	Normal 1			Reversed 1			Normal 2			Reversed 2		
	r	G	δ	r	G	δ	r	G	δ	r	G	δ
P1	0.99	0.64	5.11	0.44	−0.33	29.97	0.98	0.56	5.70	0.27	0.15	23.74
P2	0.98	0.42	4.17	0.89	0.44	10.65	—	—	—	—	—	—
P3	0.98	0.50	4.56	0.82	−0.52	16.94	0.98	0.54	4.60	0.78	0.41	15.07
P4	1.00	0.53	2.36	0.95	−0.65	10.40	0.98	0.64	6.76	0.39	0.22	24.01
P5	0.92	0.51	9.31	0.45	0.18	16.79	0.99	0.49	3.16	0.97	0.50	5.60
P6	0.99	0.50	3.99	0.13	−0.05	16.98	0.98	0.66	6.74	0.86	0.50	13.89
P7	1.00	0.52	2.41	0.07	0.00	36.49	—	—	—	—	—	—
P8	0.99	0.65	3.36	0.99	0.74	5.20	0.98	0.61	5.16	0.98	0.70	6.32
P9	0.99	0.62	4.24	0.99	0.64	4.38	0.80	0.43	14.66	0.77	0.41	15.52
P10	0.98	0.48	4.32	0.98	0.54	4.64	0.98	0.57	5.05	0.96	0.54	7.81

Rows contains for each participant (P1–P10) the values of coefficients of the linear fit according to the listening conditions (r: Pearson’s correlation; G: gain; δ: SD of residuals).

The results before training are similar to those we found in the dynamic listening conditions of Experiment 1. In Normal 1 the performance fits with a linear regression. In Reversed 1, by contrast, the regression coefficients show a worse correlation, higher levels of δ, and negative G values. To compare the first two conditions (Normal 1 vs. Reversed 1) we perform a repeated-measures ANOVA on mean localization error using listening condition and source position as factors. There is a main effect of listening condition (F_(1, 7)_ = 29.7; p < 0.03; η^2^ = 0.499). Errors in Reversed 1 are significantly higher than in Normal 1. There is also a significant interaction between listening condition and source position (F_(6, 42)_ = 445.01, p < 0.003, η^2^ = 0.362). Pairwise post hoc comparison showed that localization errors are significantly higher in Reversed 1 than in Normal 1 in Lateral positions, right and left (both p < 0.001), in Right Intermediate Lateral (p < 0.009) and Left (p < 0.0001), and in the position Ahead to the Right (p < 0.004).

After training, in Normal 2, participants’ performance shows a good linear relationship. In Reversed 2, their performance also fits with a linear regression. Although its precision is variable, the G value of all is positive, that is, most of the responses are in the hemifield where the source is located (Fig. 4 and Table 2). To compare the last 2 conditions (Normal 2 vs Reversed 2) we perform a repeated-measures ANOVA on mean localization error taking listening condition and source position as factors. Regarding the listening condition, there is no significant effect (F_(1, 7)_ = 0.161; p = 0.7003; η^2^ = 0.022). Errors in Reversed 2 are not statistically different that in Normal 2. However, interaction between condition and position show significant effects (F_(6, 42)_ = 2.3567, p < 0.047, η^2^ = 0.251). Post hoc analysis indicates significantly higher errors in Reversed 2 than in Normal 2 only in Lateral positions, both right (p < 0.045) and left (p < 0.0001). After training, performance in Normal 2 and in Reversed 2 was different only in the extreme lateral positions.

Learning depends on source position. To better understand this dependence, we examine the exploration patterns made by participants. For this, we need an estimation of the amount of motor activity that is relevantly put to use in the localization task. As an approximation, we count the number of crossings that participants make over the direction of the active source. A crossing happens when the center of the face passes through the line connecting the source and the center of the head. This number serves to estimate the degree of “sensorimotor engagement”, i.e., the sensorimotor interaction between a participant and the object or space she is exploring. We assume that a higher number of crossings corresponds to a stronger sensorimotor engagement.

**Figure 5:**
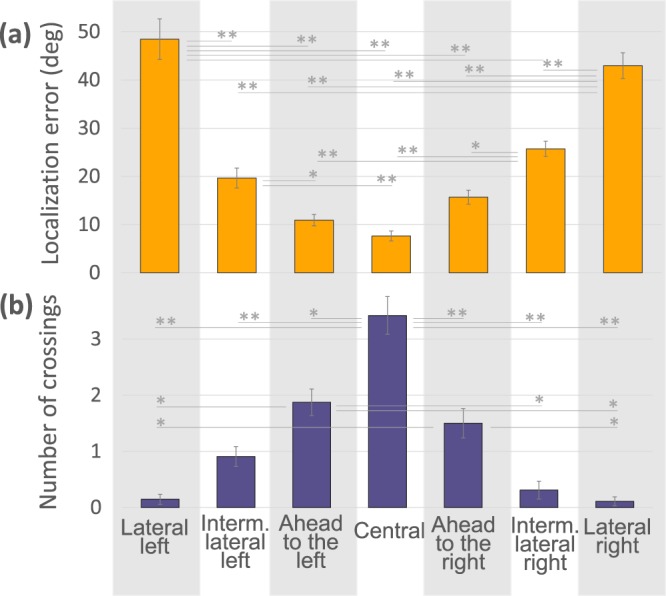
Localization errors and number of crossings in reversed conditions during training and subsequent trials. (**a**) Mean (±SEM) localization error according to source positions. (**b**) Mean (±SEM) number of crossings according to source positions. In the most peripheral positions localization errors are greater and fewer crossings are made compared to the central positions (*p < 0.05, **p < 0.001).

Considering all the reversed listening condition trials in which participants received feedback, we collapse the data of Reversed 2 and the training phase for the 5 participants who passed it satisfactorily. We analyze localization errors and number of crossings according source position (Fig. 5) and perform repeated-measures ANOVA of mean localization error taking source position as a factor. The effect of source position is significant (F_(6, 24)_ = 10.068, p < 0.0001, η^2^: 0.715). Pairwise post hoc comparison shows that the errors in Lateral positions are significantly higher than in the rest of the positions (p < 0.0001 all comparisons). Errors in Left Intermediate Lateral are higher than in Ahead to the Left (p < 0.03) and the Central positions (p < 0.008). Errors in the Right Intermediate Lateral are higher than in the Ahead to the Right (p < 0.04), Ahead to the Left (p < 0.009), and Central (p < 0.002) positions. Finally, errors in Ahead to the Right and Ahead to the Left, are higher than in the central position, the easiest position. We also compare with a repeated-measures ANOVA the number of crossings per trial using source position as a factor. There is also a significant effect on the number of crossings (F_(6, 24)_ = 10.068, p < 0.0001 η^2^: 0.715). Post hoc analysis shows that in the Central position participants carry out more crossings than in the rest of the positions (p < 0.0001 compared to both Lateral and Right Intermediate Lateral, p < 0.002 compared to Left Intermediate Lateral, p < 0.008 compared to Ahead to the Left, and p < 0.01 compared to Ahead to the Right). In the Ahead to the Left position participants make more crossings than in the Lateral position (p < 0.03 compared to Right and p < 0.04 compared Left) and Right Lateral Intermediate (p < 0.04). Also in the Ahead to Left they perform more crossings than in the Lateral Right (p < 0.03) and Lateral Left positions (p < 0.04). In the Central position participants show the best performance and the highest number of crossings. In contrast, in the Lateral positions, the most difficult to locate with accuracy, participants make the least amount of crossings. In other words, learning is more effective in positions that are more actively engaged by the participants.

#### Motor trajectories

**Figure 6:**
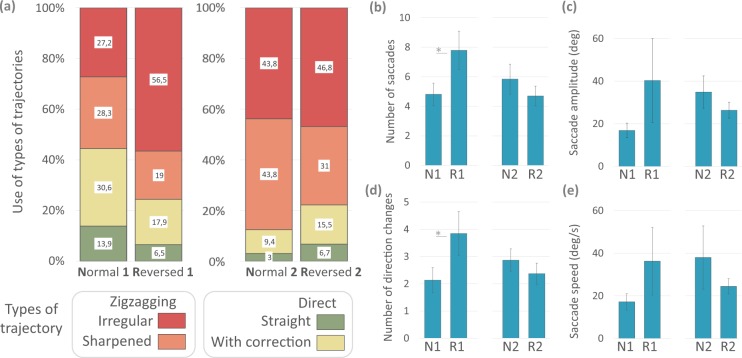
Trajectories and indexes of head movements in Experiment 2. (**a**) Percentage of use of each type according to listening condition. On the right, the plots show the mean values (±SEM) of different parameters of head saccades according to listening condition (N1: Normal 1; R1: Reversed 1; N2: Normal 2; R2: Reversed 2): (**b**) shows the number of head saccades, (**c**) their amplitude, (**d**) the number of direction changes, and (**e**) the speed (*p < 0.05).

We classify head movement trajectories as in Experiment 1. We perform Wilcoxon tests to compare the use of each trajectory and t-test to compare kinematics indexes in Normal 1 vs Reversed 1. The results are similar to those previously observed; the reversed listening condition seems to force the participants to modify their normal exploration patterns (Fig. 6a). The same comparisons between Normal 2 vs. Reversed 2 do not show significant differences for the type of trajectories and for kinematics indexes (Fig. 6b–e). After training, participants make similar use of exploration strategies in the two listening conditions.

#### Participants’ reports

Most participants reported using an “analytical” strategy in condition Reversed 2. For example, moving the head in search of a position with similar sound intensity in both ears: “*I was trying to get the same sound in both ears*” (Participant 4), “*I tried to hear the same level of sound in both ears, to get the same level of sound pressure*” (Participant 8). Some participants mentioned that when facing the sound, they heard changes that helped them ascertain that the source was directly ahead. For example: “*When [the source] was in front of me, I heard something strange like a ‘tic’. I heard it at the back of my neck, with the same level in both ears*.” (Participant 5), “*A particular sound like ‘prr … ‘ was generated when you were facing the direction of the sound [source]*.” (Participant 9). These experiences cannot be attributed to a malfunctioning of the pseudophone since all validations on the device (objective and subjective) showed a proper operation throughout the experiment.

#### Particular learning paths

We encountered a wide variety in learning. Three participants in Experiment 1 and another three in Experiment 2 were able to correctly locate the sound source in the reversed listening condition without feedback. Their sensorimotor strategies seemed already accommodated to coping with the reversed listening condition. Something similar was observed by Held^13^. In these cases, participants found from the beginning an effective way of locating the sound source in either condition. It usually involved moving the head from one side to the other and stopping it in the place where the sound level was equal in both ears.

In contrast, two participants in Experiment 2 failed to locate sounds in the reversed listening condition—one (P7) failed to complete the requirements to pass the training phase and the other (P2) requested to leave the experiment after completing a third of the trials in the last 2 conditions. Until that moment she had failed in all the reversed listening trials of that series. Her strategy in the previous reversed trials was to move in the direction opposite to where the sound seemed to come from at the start of the trial. This strategy no longer served her after training because normal and reversed trials were presented randomly.

The rest of the participants experienced a gradual process of learning. The case of Participant P1 serves as an example (Table 2). Her overall performance was similar to the average of all the participants. Her relatively low and highly variable performance in Reversed 2 can be better understood by considering her localization errors at the Lateral positions, which were high and variable. In the other positions her performance was considerably better. This is consistent with her report:

*“[at the beginning] I sensed the sound going behind the head. I gave my response but I didn’t feel that I was facing the source. At some point, moving my head randomly, I felt that I was facing [the source] and I realized that was the same feeling as when I was facing a sound source normally. (…) First, I was trying for the sound to reach both ears equally. Then, in the last part, I felt it clearly as a sensation, not just auditory. I don’t know how to explain it, it was like a sensation in my head that told me: yes, there it is. This happened with these [speakers] at the front. In those others [lateral speakers] I realized I was not right.”*

**Figure 7:**
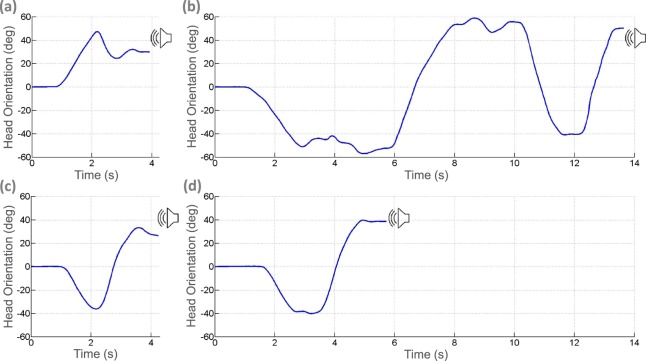
Examples of head trajectories for Participant P1 with a sound source in the Right Lateral Intermediate position (45 deg) according to listening condition: (**a**) Normal 1, (**b**) Reversed 1, (**c**) Normal 2, and **(d**) Reversed 2. The plot corresponds to 4 individual trials where vertical axis represents the head orientation and horizontal axis time.

According to P1, she stopped experiencing the sensation of strangeness in the reversed listening conditions. In the central positions she began to perceive the sound source in front of her without attending to the pseudophone state. In this region, her strategy seemed to go from analytical to direct. In the Lateral positions her responses remained tentative. These considerations help us understand the plots in Fig. 7. They correspond to head movements made by P1 in the different listening conditions. While the ensembles of trials before training and after training are not directly comparable (due to the randomization condition and feedback in the last conditions), it is illustrative to compare individual trajectories in the normal and reversed conditions before and after training. In all cases the sound source was in the Right Intermediate Lateral position and it was found successfully. There is a remarkable difference between trajectories before and after training. The participant performs a more complex scan in the reversed condition before training. After training, the trajectories in both listening conditions are similar. As mentioned in her report, the participant no longer needed to perform an elaborate strategy during reversed listening.

## Discussion

Experiment 1 shows that, on first time use, the pseudophone tends to induce a strong disturbance in the ability to find sounds. The disruptive effect is not simply due to the acoustic field reversion. To be sure, as the passive reversed listening condition demonstrates, participants perceive short sound pulses in the hemifield opposite to their actual source. But in the dynamic listening condition, where participants can move their heads freely and sound is continuous, reversion produces unpredictable changes in the accuracy of the responses. It also alters exploration strategies and gives rise to experiences of disorientation and strange phenomena such as the impression that fixed sound sources are moving or jumping positions. Similar effects have been reported^8,10,11^ and analogous phenomena are well-documented in studies of vision reversion/inversion^1,7^. According to the sensorimotor approach to perception^24,25^, reversion generates a breakdown in the sensorimotor contingencies that the person masters. Changes in the sound patterns generated by movement go against expectations and make habitual ways of exploring the environment ineffective.

In Experiment 2, most participants show evidence of learning to find sounds in the reversed listening condition. At the end of the experiment, their performance does not differ from the normal condition for most source positions except the extreme lateral ones. Head movements have similar kinematic features in both listening conditions. Participants in the reversed condition cease reporting the experience of movement-induced instability and some report an experience of “correctness” or certainty when adequately orienting towards the source. Results show that participants develop new sensorimotor strategies to find sound sources. These strategies change in a manner characteristic of many sensorimotor learning processes. At the beginning they carry a large monitoring load on each action, but over time movements become more direct and unreflective^25^.

Adaptation does not follow a unique pattern. There are significant individual differences between the participants. Although the majority can adapt with the feedback provided, 3 of them could do it without any feedback and 2 could not adapt at all. In part, this can be explained by individual variability in adapting hearing skills. Large individual differences have been revealed in other adaptation experiments in sound localization^32^. Similar variability has been observed in the learning of auditory spatial abilities in people who have suffered an abrupt change in their sensitivity such as the loss of hearing in one ear^33^ or the use of cochlear implants^34^.

Participants show better performance in the frontal positions than in the extreme lateral ones (close to +/− 67.5 deg). This is consistent with evidence that when adaptation occurs it is seldom spatially homogeneous^35,36^. Why is it harder to adapt to locating sound sources at the periphery? One possible answer is that the difficulty is determined by differences in prior spatial hearing sensitivity. Head orientations with higher sensitivity in normal listening conditions correspond to positions with better adaptation. We would expect participants to maintain a similarly shaped angular distribution of errors before and after adaptation: with more accurate responses in the frontal region than in the lateral ones. However, in Reversed 2 participants do not show the same distribution of errors. Performance is similar in Normal 2 and Reversed 2 in frontal and intermediate lateral positions but different in the extreme lateral positions. In these directions participants adapt significantly less to the reversed listening condition.

This additional loss in performance can be explained by how participants actively explore the different positions, i.e., their sensorimotor engagement with the environment during adaptation^32^. According to the crossing index, participants engage the front sources more frequently than the peripheral ones. This may seem obvious since the set-up forced participants to remain seated and face the frontal region more often. Lateral head movements in relation to the torso are easier facing the frontal region than towards the sides. More motor variability provides more opportunities to learn sensory changes that are contingent on head movements. We suggest that participants are better able to generate suitable new sensorimotor patterns facing the frontal regions because these can be more easily explored, leading to a better adaptation to sound localization in these directions. The idea that as motor patterns become more difficult so does adaptation is consistent with the observation that adaptation to a given distortion is not generalizable to unpracticed sensorimotor patterns^37^.

Extrapolating to the more general case of perceptual development, we can hypothesize that typical differential sensitivity to sound localization in the horizontal plane is itself in part a result of bodily constraints on active exploration. In everyday life we interact much more frequently with frontal sources rather than peripheral ones, which leads us to learn to find them more accurately. When required, we tend to move the torso or the whole body to explore sounds arriving laterally. It would be expected that bodies with more extended movement range will in general possess a better ability for locating peripheral sounds, as in the case of the barn owl’s greater amplitude for head rotation and better sound localization sensitivity on the horizontal plane^38^.

Why do participants adapt to auditory reversion in this study but not in previous ones, even when the pseudophone is used only for short periods? Learning depends on a number of key factors: (1) performing the whole task without visual cues to avoid the ventriloquism effect, (2) working in a simplified sound environment, and (3) allowing participants to explore it with rotational head movements to generate and learn new auditory cues^39^. In previous work where participants were allowed to see during the training phase, they were trained in natural environments and complex soundscapes. In addition, they were not allowed to freely explore the experimental environment. Although many auditory skills are calibrated with vision^40,41^ it seems that managing without vision in cases like these experiments enables better auditory spatial learning. For example, Carlile, *et al*.^20^ compared the effect of using different types of feedback on accommodation to auditory spectral perturbation generated by the use of ear molds. The results showed that participants significantly improve their abilities to find sounds when they have auditory-motor feedback compared to auditory-visual feedback. Similarly, studies of adaptation to non-individualized Head Related Transfer Functions in virtual environments demonstrate the efficacy of using sensorimotor information to improve spatial hearing^42,43^. Sensorimotor-based learning helps explain the development of auditory spatial abilities in people with normal vision^20,44^ and also in blind people whose auditory skills, in some cases, are better^45,46^.

In Experiment 2 we induced active learning by randomly altering normal and reversed listening conditions between trials. Some participants develop a single sensorimotor strategy valid for the two conditions. This possibility has been suggested by James. G. Taylor in studies with radical disruptions to left/right reversed visual field^47^. It has also been shown that with disruptions that displace vision and alter the ability to execute manual tasks, participants perform similarly with and without visual displacement thanks to contextual clues that indicate the disruptive state^48–50^ or, as in this experiment, by making movements that help disambiguate the state^51,52^. Computer simulation models show that strategies that can simultaneously cope with spatially opposed sensorimotor conditions are feasible^53^.

The analysis of trajectories suggests that there may be a functional equivalence between visual saccades and the head movements participants make to locate sounds. For practical purposes the comparison is appropriate, since it allows us to analyze in detail a complex sequence of sensorimotor patterns. Other studies have described the same analogy in sound localization tasks in humans^20^, in owls^38^, studying echolocation abilities in humans^54^ and bats^55,56^, and with the use of sensory substitution devices in humans^57^. It would be interesting to study if there are common mechanisms in different sensory modalities that account for the emergence of saccade-like patterns.

If we compare the two experiments, we observe different strategies under two apparently similar conditions: Dynamic Normal in Experiment 1 and Normal 1 in Experiment 2. Technically, the conditions themselves are the same, they have the same set-up, the same stimuli and the same task. However, they differ in the number of trials and the order of administration. The order of presentation of conditions influences the trajectories. In Experiment 1, before the Dynamic Normal condition, participants performed the test in the Normal Passive condition, where most trajectories were direct, which probably favored direct response strategies. While in Experiment 2, condition Normal 1 took place at the beginning with no prior influence and participants opted for zigzagging trajectories. Despite this difference, the values of the linear regression coefficients and the magnitude of the location errors are similar between both conditions.

The experimental design has limitations. It was necessary to randomly alternate the state of the pseudophone in the final phase in order to avoid stereotypical or pre-planned responses. But this has the disadvantage of impeding the evaluation of adaptation aftereffects. In the future it will be informative to evaluate the degree of adaptation achieved during the reversed listening condition by comparing it to an immediately subsequent passive condition of normal listening. Also, we could investigate learning improvement and changes in sensorimotor strategies in more detail through more extensive training phases. Finally, it will be interesting to evaluate if the results generalize to situations with multiple and varying sound stimuli, different positions, and more complex sound scenarios.

## Data Availability

Datasets generated for this study are available from the Open Science Framework repository (https://osf.io/mu58j/?view_only=57d1a919f53344b9b605db23ce6a1bcc).

## References

[1] Kohler I (1962). Experiments with goggles. Scientific American..

[2] Held R, Freedman SJ (1963). Plasticity in human sensorimotor control. Science..

[3] O’Regan JK, Noë A (2001). A sensorimotor account of vision and visual consciousness. Behavioral and Brain Sciences.

[4] Stoffregen TA, Bardy BG (2001). On specification and the senses. Behavioral and Brain Sciences.

[5] Stratton GM (1897). Vision without inversion of the retinal image. Psychological review.

[6] Sugita Y (1996). Global plasticity in adult visual cortex following reversal of visual input. Nature.

[7] Degenaar J (2014). Through the inverting glass: first-person observations on spatial vision and imagery. Phenomenology and the Cognitive Sciences.

[8] Young PT (1928). Auditory localization with acoustical transposition of the ears. Journal of Experimental Psychology.

[9] Willey CF, Inglis E, Pearce CH (1937). Reversal of auditory localization. Journal of Experimental Psychology.

[10] Ohtsubo H, Teshima T, Nakamizo S (1982). Effects of head movements on sound localization with an electronic pseudophone. Japan Psychological Research.

[11] Hofman PM, Vlaming MS, Termeer PJ, Van Opstal AJ (2002). A method to induce swapped binaural hearing. Journal of neuroscience methods.

[12] Welch, R. B. Perceptual modification: Adapting to altered sensory environments (Academic Press, 1978).

[13] Held R (1955). Shifts in binaural localization after prolonged exposures to atypical combinations of stimuli. The American journal of psychology..

[14] Middlebrooks JC, Green DM (1991). Sound localization by human listeners. Annual review of psychology.

[15] Blauert, J. Spatial hearing: the psychophysics of human sound localization (MIT press, 1997).

[16] Goossens HHLM, Van Opstal AJ (1999). Influence of head position on the spatial representation of acoustic targets. Journal of neurophysiology.

[17] Carlile S, Leung J (2016). The perception of auditory motion. Trends in hearing.

[18] Froese, T. & González-Grandón, X. How passive is passive listening? Toward a sensorimotor theory of auditory perception. *Phenomenology and the Cognitive Sciences*. 1–33 (2019).

[19] Perrott DR, Saberi K, Brown K, Strybel TZ (1990). Auditory psychomotor coordination and visual search performance. Perception & Psychophysics..

[20] Carlile S, Balachandar K, Kelly H (2014). Accommodating to new ears: the effects of sensory and sensory-motor feedback. The Journal of the Acoustical Society of America.

[21] Brimijoin WO, McShefferty D, Akeroyd MA (2010). Auditory and visual orienting responses in listeners with and without hearing-impairment. The Journal of the Acoustical Society of America.

[22] Mueller MF, Meisenbacher K, Lai WK, Dillier N (2014). Sound localization with bilateral cochlear implants in noise: How much do head movements contribute to localization?. Cochlear Implants International..

[23] Pastore MT, Natale SJ, Yost WA, Dorman MF (2018). Head movements allow listeners bilaterally implanted with cochlear implants to resolve front-back confusions. Ear and hearing.

[24] Noë, A. Action in perception (MIT Press, 2004).

[25] Di Paolo, E., Buhrmann, T. & Barandiaran, X. Sensorimotor life: An enactive proposal (Oxford University Press, 2017).

[26] Aytekin M, Moss CF, Simon JZ (2008). A sensorimotor approach to sound localization. Neural Computation.

[27] Gilberto G (2016). Inversión en la percepción binaural: diseño y validación de un pseudófono. Mecánica Computacional..

[28] Pedersen, J. A. & Jorgensen, T. Localization performance of real and virtual sound sources. In New Directions for Improving Audio Effectiveness. 29-1–29-30 (RTO, 2005).

[29] Lunati V, Podlubne A, Bermejo F, Arias C (2013). Análisis de fijaciones en movimientos para localización y reconocimiento auditivo de objetos. Mecánica Computacional..

[30] Lewald J, Dörrscheidt GJ, Ehrenstein WH (2000). Sound localization with eccentric head position. Behavioural brain research.

[31] Thurlow WR, Mergener JR (1970). Effect of stimulus duration on localization of direction of noise stimuli. Journal of speech and hearing research.

[32] Carlile S (2014). The plastic ear and perceptual relearning in auditory spatial perception. Frontiers in Neuroscience..

[33] Firszt JB, Reeder RM, Dwyer NY, Burton H, Holden LK (2015). Localization training results in individuals with unilateral severe to profound hearing loss. Hearing research.

[34] Tyler RS (2010). An attempt to improve bilateral cochlear implants by increasing the distance between electrodes and providing complementary information to the two ears. Journal of the American Academy of Audiology.

[35] Shinn-Cunningham BG, Durlach NI, Held RM (1998). Adapting to supernormal auditory localization cues. II. Constraints on adaptation of mean response. The Journal of the Acoustical Society of America..

[36] Welch, R. B. (1986). Adaptation of space perception in Handbook of perception and human performance (Eds. Boff, K. R. Kaufman, L. &. Thomas, J. P.) 24.1–24.45 (John Wiley and Sons, 1986).

[37] Bock O (2013). Basic principles of sensorimotor adaptation to different distortions with different effectors and movement types: a review and synthesis of behavioral findings. Frontiers Human. Neuroscience..

[38] Knudsen EI, Konishi M (1979). Mechanisms of sound localization in the barn owl (Tyto alba). Journal of Comparative Physiology.

[39] Honda A (2013). Effects of Head Movement and Proprioceptive Feedback in Training of Sound Localization. I-Perception..

[40] Witkin. HA, Wapner S, Leventhal T (1952). Sound localization with conflicting visual and auditory cues. Journal of Experimental Psychology..

[41] King AJ (2009). Visual influences on auditory spatial learning. Philosophical Transactions of the Royal Society B: Biological Sciences..

[42] Parseihian G, Katz BFG (2012). Rapid head-related transfer function adaptation using a virtual auditory environment. The Journal of the Acoustical Society of America..

[43] Majdak P, Walder T, Laback B (2013). Effect of long-term training on sound localization performance with spectrally warped and band-limited head related transfer functions. The Journal of the Acoustical Society of America..

[44] Lewald J (2007). More accurate sound localization induced by short-term light deprivation. Neuropsychologia.

[45] Röder B, Rösler F, Neville HJ (1999). Effects of interstimulus interval on auditory event-related potentials in congenitally blind and normally sighted humans. Neuroscience letters.

[46] Voss P, Gougoux F, Zatorre RJ, Lassonde M, Lepore F (2008). Differential occipital responses in early-and late-blind individuals during a sound-source discrimination task. Neuroimage.

[47] Taylor, J. G. The behavioral basis of perception. (Yale University Press, 1962).

[48] Ghahramani Z, Wolpert DM (1997). Modular decomposition in visuomotor learning. Nature..

[49] Wolley DG, Tresilian JR, Carson RG, Rick S (2007). Dual adaptation to two opposing visuomotor rotations when each is associated with different regions of workspace. Experimental Brain Research..

[50] Seidler RD, Bloomberg JJ, Stelmach GE (2001). Context-dependent arm pointing adaptation. Behavioural Brain Research..

[51] Howard IS, Ingram JN, Franklin DW, Wolpert DM (2012). Gone in 0.6 seconds: the encoding of motor memories depends on recent sensorimotor States. Journal of Neuroscience..

[52] Hirashima M, Nozaki D (2012). Distinct motor plans form and retrieve distinct motor memories for physically identical movements. Current Biology..

[53] Izquierdo-Torres, E. & Di Paolo, E. A. Is an embodied system ever purely reactive? In Advances in Artificial Life (eds. Capcarrere M. S. *et al*.) 252–261 (Springer, 2005).

[54] Milne JL, Goodale MA, Thaler L (2014). The role of head movements in the discrimination of 2-D shape by blind echolocation experts. Attention, Perception, & Psychophysics.

[55] Surlykke A, Ghose K, Moss CF (2009). Acoustic scanning of natural scenes by echolocation in the big brown bat, Eptesicus fuscus. Journal of Experimental Biology.

[56] Seibert AM, Koblitz JC, Denzinger A, Schnitzler HU (2013). Scanning behavior in echolocating common pipistrelle bats (Pipistrellus pipistrellus). PloS one.

[57] Bermejo F, Di Paolo EA, Hüg MX, Arias C (2015). Sensorimotor strategies for recognizing geometrical shapes: A comparative study with different sensory substitution devices. Frontiers in psychology..

